# Distribution and Quantitative Estimates of Variant Creutzfeldt-Jakob Disease Prions in Tissues of Clinical and Asymptomatic Patients

**DOI:** 10.3201/eid2306.161734

**Published:** 2017-06

**Authors:** Jean Y. Douet, Caroline Lacroux, Naima Aron, Mark W. Head, Séverine Lugan, Cécile Tillier, Alvina Huor, Hervé Cassard, Mark Arnold, Vincent Beringue, James W. Ironside, Olivier Andréoletti

**Affiliations:** Institut National de la Recherche Agronomique, Toulouse, France (J.Y. Douet, C. Lacroux, N. Aron, S. Lugan, C. Tillier, A. Huor, H. Cassard, O. Andréoletti);; University of Edinburgh, Edinburgh, Scotland, UK (M.W. Head, J.W. Ironside);; Animal and Plant Health Agency, Loughborough, UK (M. Arnold); Institut National de la Recherche Agronomique, Jouy-en-Josas, France (V. Beringue)

**Keywords:** prions and related diseases, Creutzfeldt-Jakob disease, variant Creutzfeldt-Jakob disease, vCJD, variant Creutzfeldt-Jakob disease prions, protein misfolding cyclic amplification, variant infectivity, intracerebral inoculation, tissues, clinical patients, asymptomatic patients, meningitis/encephalitis, public health, Scotland, United Kingdom

## Abstract

In the United-Kingdom, ≈1 of 2,000 persons could be infected with variant Creutzfeldt-Jakob disease (vCJD). Therefore, risk of transmission of vCJD by medical procedures remains a major concern for public health authorities. In this study, we used in vitro amplification of prions by protein misfolding cyclic amplification (PMCA) to estimate distribution and level of the vCJD agent in 21 tissues from 4 patients who died of clinical vCJD and from 1 asymptomatic person with vCJD. PMCA identified major levels of vCJD prions in a range of tissues, including liver, salivary gland, kidney, lung, and bone marrow. Bioassays confirmed that the quantitative estimate of levels of vCJD prion accumulation provided by PMCA are indicative of vCJD infectivity levels in tissues. Findings provide critical data for the design of measures to minimize risk for iatrogenic transmission of vCJD.

Prion diseases, or transmissible spongiform encephalopathies (TSEs), are fatal neurodegenerative disorders that occur naturally in sheep (scrapie), cattle (bovine spongiform encephalopathy [BSE]), and humans (Creutzfeldt-Jakob disease [CJD]). A key event in the pathogenesis of TSEs is the conversion of the normal cellular prion protein (PrP^C^, encoded by the *PRNP* gene) into an abnormal disease-associated isoform (PrP^Sc^) in tissues of infected animals. PrP^C^ is completely degraded after controlled digestion with proteinase K in the presence of nondenaturing detergents. In contrast, PrP^Sc^ is N terminally truncated under the same conditions, resulting in a proteinase K–resistant prion (PrP^res^) (*1*).

In 1996, a new form of CJD, termed variant CJD (vCJD) was identified in the United Kingdom. vCJD is believed to result from zoonotic transmission of the BSE agent, probably as a consequence of dietary exposure to BSE-contaminated meat products (*2*,*3*). The total number of clinical cases of vCJD thus far identified is limited (227 patients worldwide at the time of writing this article). However, the estimated prevalence of asymptomatic vCJD in populations exposed to the BSE agent is uncertain (*4*).

In the United Kingdom, 32,441 appendix samples collected during surgery from patients born during 1941–1985 have been tested for abnormal prion protein accumulation by using immunohistochemical analysis. This study reported a vCJD prevalence estimate of 1/2,000 in persons in these age cohorts (95% CI 1/3,500–1/1,250) (*5*). No comparable data are available concerning the prevalence of asymptomatic vCJD in other countries, although BSE exposure is known to have occurred in several countries in continental Europe, as judged by cases of vCJD that are not attributable to exposure in the United Kingdom (http://www.cjd.ed.ac.uk/documents/worldfigs.pdf).

Over the past 2 decades, several studies have reported on the distribution of the vCJD agent in tissues of infected patients (*6*–*8*). Most of these studies did not detect the vCJD agent outside the nervous system (central, peripheral, and autonomic) and lymphoid tissues. However, the sensitivity of detection techniques for PrP^res^ used in these investigations was limited.

Protein misfolding cyclic amplification (PMCA) is believed to mimic prion replication in vitro, but in an accelerated form, which enables amplification of minute amounts of PrP^Sc^ and prion infectivity (*9*). In PMCA, a PrP^C^-containing substrate is combined with a seed that might contain otherwise undetectable amounts of PrP^Sc^. After repeated cycles of incubation and sonication, the amount of PrP^Sc^ increases to levels at which they can be detected by using conventional biochemical techniques. Recently, our group and others have shown that PMCA can detect endogenous vCJD agent in patient biologic fluids such as urine and blood (*10*,*11*).

In this study, we evaluated the relative sensitivity of PMCA versus that of bioassay in mice for detection of the vCJD agent. We estimated by using PMCA the level of vCJD prions in 21 tissues collected from 4 patients who died of symptomatic vCJD and from a patient with asymptomatic vCJD. We also determined whether vCJD prion levels, as estimated by using PMCA, were consistent with infectious titers, as estimated by bioassay with transgenic mice.

## Methods

### Ethics Statements

All animal experiments were performed in compliance with institutional and French national guidelines and in accordance with the European Community Council Directive 86/609/EEC. Animal experiments that were part of this study (national registration no. 01734.01) were approved by the local ethic committee of the Ecole Nationale Vétérinaire de Toulouse (Toulouse, France). Mouse inoculations were performed under anesthesia with isofulorane. Mice that displayed clinical signs of disease were anesthetized with isofluorane before being humanely killed by inhalation of CO_2_.

Human samples were obtained from the United Kingdom National CJD Research and Surveillance Unit Brain and Tissue Bank, which is part of the Medical Research Council Edinburgh Brain Bank (Edinburgh, Scotland, UK). Tissue samples were pseudo-anonymized by using a Brain Bank reference number. All case-patients in the United Kingdom provided informed consent. Use of samples in this study was approved by the East of Scotland Research Ethics Service for the Edinburgh Brain Bank (16/ES/0084).

### vCJD and Control Patients

We investigated tissues from 4 clinical vCJD case-patients (vCJD-1–vCJD-4) and 1 asymptomatic person with vCJD who had received a transfusion of packed erythrocytes from a donor who subsequently died from vCJD (*12*). Tissues from 2 non–vCJD–affected patients were used as controls. For case-patients who provided appropriate consent, the entire *PRNP* gene coding sequence was established to exclude pathogenic mutations in this gene (*13*,*14*).

### Mouse Bioassays

Bioassays were performed by using mice expressing bovine PrP (tgBov-tg110) as described (*15*,*16*). These mice were observed daily and their neurologic status was assessed weekly. When clinically progressive TSE symptoms were evident, or at the end of their lifespan, the animals were euthanized. Survival time was expressed as the mean ± SD days postinoculation of mice positive for PrP^res^. For mice that showed no clinical signs, they were humanely killed at the end of their natural lifespan (600–800 days). In these instances, incubation periods are reported as >600 days postinoculation, which corresponded to survival time observed for >3 of 6 mice.

### Estimation of Infectious Prion Titers

We estimated infectious titer in a reference 10% (wt/vol) frontal cortex homogenate from a clinical vCJD patient by using endpoint titration (intracerebral route) in tgBov mice. Infectious titer (50% lethal dose/g intracerebral in tgBov mice) was estimated by using the Spearman method.

The titer of prion infectivity in vCJD–affected patient bone marrow samples was estimated by using the method of Arnold et al. (*17*). This method uses the probability of survival (attack rate at each dilution) and the individual mouse incubation periods at each dilution to estimate infectious load and is thus able to provide more accurate estimation of titer than using either attack rate or incubation period data alone.

### PMCA Reactions

A transgenic mouse line that expresses ovine A_136_R_154_Q_171_ PrP variant PrP^C^ (tgShXI) was used to prepare the PMCA substrate as described (*18*,*19*). PMCA amplification was performed as described (*11*). Each PMCA experiment included a reference vCJD sample (10% brain homogenate) as a control for the amplification efficiency. Unseeded controls (1 unseeded control for 8 seeded reactions) were also included in each experiment. For each tested dilution of each sample, >4 replicates were tested in 2 independent experiments. For each sample, the highest dilution showing >50% of positive replicates (presence of detectable PrP^res^ in the reaction as assessed by using Western blotting) was determined.

### Detection of Abnormal PrP by Western Blotting and Paraffin-Embedded Tissue Blotting

Extraction of proteinase K–resistant abnormal PrP and Western blotting were performed as described (*11*). Immunodetection was performed by using 2 PrP-specific monoclonal antibodies, Sha31 (1 μg/mL) (*20*), and 12B2 (4 μg/mL) (*21*), which recognize amino acid sequences YEDRYYRE (145–152), and WGQGG (89–93), respectively. Paraffin-embedded tissue blotting was performed as described (*22*,*23*).

## Results

### Sensitivity of vCJD Agent Detection by PMCA and Bioassay

**Figure 1:**
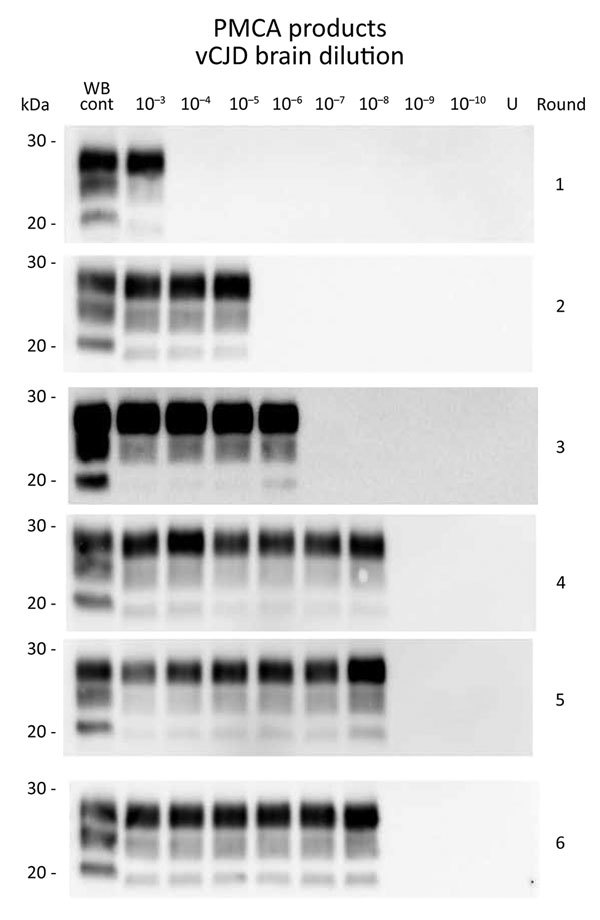
Western blots of variant Creutzfeldt-Jakob disease (vJCD) proteinase K–resistant prions (PrP^res^) analyzed by protein misfolding cyclic amplification (PMCA) in tissues of clinical and asymptomatic patients. PMCAs were seeded with a 10-fold serial dilutions of a reference vCJD brain homogenate (10% wt/vol, 10^−2^–10^−10^ dilutions). This homogenate had been endpoint titrated by bioassay in bovine prion (PrP)–expressing mice (tgBov, intracerebral route, 10^7.7^ 50% lethal dose/g). PMCA substrate was prepared by using brains from transgenic mice overexpressing the ARQ variant of sheep prion protein. An unseeded (lane U) reaction was included as a specificity control. PMCAs were subjected to 6 rounds of amplification, each composed of 96 cycles (sonication for 10 s and incubation for 14.5 min at 39.5°C) in a Qsonica700 Sonicator (Qsonica LLC, Newtown, CT, USA). After each round, reaction products (1 volume) were mixed with fresh substrate (9 volumes) to seed the following round. Part of the same product was analyzed by Western blotting for abnormal PrP^res^ (Sha31 antibody epitope YEDRYYRE). A sheep scrapie sample (WB cont) was included as a control on each gel. WB, Western blot.

To determine the relative sensitivity of PMCA, we retitrated a reference sample (10% cerebral cortex homogenate from a vCJD-affected patient) that had previously undergone endpoint titration (IC inoculation route; Table 1) in bovine PrP–expressing mice (tgBov). Amplification of a 10-fold serial dilution of this sample (6 individual replicates/dilution point) demonstrated that 4 PMCA rounds (24 hours/round, i.e., 96 h) were sufficient to reach the maximum sensitivity level of the assay. Additional PMCA rounds did not improve the analytical sensitivity of the assay or the number of positive replicates (Table 2; Figure 1). On the basis of these results, we estimated by using the Spearman method that the seeding activity of the isolate was 10^11^ 50% seeding activity/per g. Bioassay endpoint titration data for the same sample in tgBov mice showed an infectious titer of 10^7.7^ LD_50_/g. When we took into account the 4-fold lower amount of material used to seed the PMCA reaction compared with material used in mouse inoculations, we found that the PMCA protocol used was 465 times more sensitive than the bioassay of tgBov mice for detection of vCJD prions.

**Table 1:** Endpoint titration of reference sample from a patient with vCJD in tgBov mice expressing bovine prion protein*

Dilution	Transmission in tgBov mice	
	No. positive mice/no. tested	Mean ± SD, incubation, d
Undiluted	6/6	249 ± 2
10^−1^	6/6	283 ± 15
10^−2^	6/6	316 ± 21
10^−3^	6/6	342 ± 10
10^−4^	6/6	453 ± 66
10^−5^	2/6	479, 495†
10^−6^	1/6	502†
10^−7^	0/6	>700

*A 10% (wt/vol) homogenate was prepared by using frontal cortex from a clinically affected patient with vCJD. Groups of 6 tgBov mice were inoculated intracerebrally with 20 μL of serial 10-fold dilutions of this homogenate. Mice were considered positive when abnormal prion protein deposition was detected in the brain. vCJD, variant Creutzfeldt-Jakob disease.  †Dilutions at which <50% of mice were positive.

**Table 2:** Endpoint titration of PMCA seeding activity in a reference brain sample from a patient with vCJD*

Amplification round	Reference vCJD 10% brain homogenate dilution series, no. of positive PMCA reactions/no. reactions conducted								
	10^−2^	10^−3^	10^−4^	10^−5^	10^−6^	10^−7^	10^−8^	10^−9^	10^−10^
1	6/6	6/6	0/6	0/6	0/6	0/6	0/6	0/6	0/6
2	6/6	6/6	5/6	3/6	0/6	0/6	0/6	0/6	0/6
3	6/6	6/6	6/6	6/6	3/6	0/6	0/6	0/6	0/6
4	6/6	6/6	6/6	6/6	6/6	5/6	2/6	0/6	0/6
5	6/6	6/6	6/6	6/6	6/6	5/6	2/6	0/6	0/6
6	6/6	6/6	6/6	6/6	6/6	5/6	2/6	0/6	0/6

*A 10% (wt/vol) homogenate was prepared by using frontal cortex from a symptomatic patient with vCJD (same homogenate as in Table 1). Samples were serially diluted 10-fold (10^−2^–10^−10^) before being used to seed PMCA reactions. Six individual replicates of each sample dilution were tested. PMCA substrate was prepared by using brains from transgenic mice overexpressing the ARQ variant of sheep prion protein. PMCAs were subjected to 6 rounds of amplification, each composed of 96 cycles (sonication for 10 s and incubation for 14.5 min at 39.5°C) in a Qsonica700 Sonicator (Qsonica LLC, Newtown, CT, USA). After each round, reaction products (1 volume) were mixed with fresh substrate (9 volumes) to seed the following round. Part of the same product was analyzed by Western blotting for abnormal PrP^res^ by using Sha31 antibody epitope YEDRYYRE. Values are number of PrP^res^ Western Blot–positive replicates corresponding to each round and each dilution. PMCA, protein misfolding cyclic amplification; PrP^res^, proteinase K–resistant prion; vCJD, variant Creutzfeldt-Jakob disease.

### PMCA for Control and vCJD Patients

**Figure 2:**
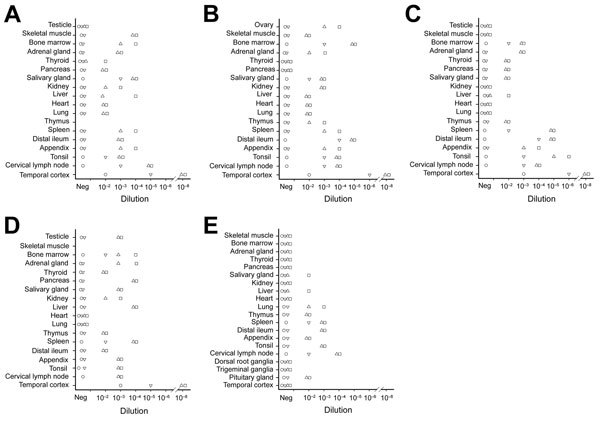
Protein misfolding cyclic amplification (PMCA) of peripheral tissues from patients with variant Creutzfeldt-Jakob disease (vCJD). PMCA reactions were seeded with a 10-fold dilution series of vCJD tissue homogenates (10^−2^–10^−9^) obtained postmortem from CJD-infected patients. At least 4 replicates of each sample dilution were tested in 2 independent PMCA experiments. Patients vCJD-1 (A), vCJD-2 (B), vCJD-3 (C), and vCJD-4 (D) died of clinical vCJD. These 4 patients were infected with prions containing methionine at codon 129 of the *PRNP* gene (homozygote). Patient vCJD-A (E) died during an asymptomatic or preclinical stage of the disease. This patient was infected with a prion containing methionine/valine at codon 129 of the *PRNP* gene (heterozygote). PMCA substrate was prepared by using brains from transgenic mice overexpressing the ARQ variant of sheep prion protein. Unseeded reactions and a reaction seeded with tissues from 2 non–vCJD-infected control patients (NC-1 and NC-2; Table 3) were included as specificity controls. PMCAs were subjected to 4 rounds of amplification, each composed of 96 cycles (sonication for 10 s and incubation for 14.5 min at 39.5°C) in a Qsonica700 Sonicator (Qsonica LLC, Newtown, CT, USA). After each round, reaction products (1 vol) were mixed with fresh substrate (9 vol) to seed the following round. Part of the same product was analyzed by Western blotting for abnormal proteinase K–resistant prions (PrP^res^) (Sha31 antibody epitope YEDRYYRE). For each round, the highest dilution showing a positive Western blot result in at least half of the replicates tested is indicated. Circles, round 1; ∇ round 2; Δ, round 3; squares, round 4. Neg, negative.

We complied basic demographic data for vCJD and control patients (Table 3). A 10-fold dilution series of 10% homogenates from the vCJD–affected and non–vCJD–affected control patients was prepared, and this series was subjected to 4 rounds of PMCA. Amplification products from each round were tested for PrP^res^ using by Western blotting (Table 4; Figure 2).

**Table 3:** Characteristics of 5 patients with vCJD and 2 controls in study of distribution and quantitative estimates of variant prions in tissues*

Patient identification no.	Diagnosis	Sex	Year of death	Age, y, at death	Disease duration, mo	*PRNP* gene codon 129	*PRNP* gene mutations
vCJD-1	vCJD	M	1999	33	18	MM	None detected
vCJD-2	vCJD	F	2000	17	18	MM	None detected
vCJD-4	vCJD	M	2000	26	10	MM	None detected
vCJD-3	vCJD	M	2001	26	10	MM	None detected
vCJD-A	Asymptomatic vCJD	F	2004	82	NA	MV	None detected
NC-1	No CJD (tumor, infarction, ischemia)	F	2005	85	NA	MM	No consent for sequencing
NC-2	No CJD (Alzheimer’s disease, infarction, ischemia)	F	2010	80	NA	MM	None detected

*NA, not applicable; vCJD, variant Creutzfeldt-Jakob disease.

**Table 4:** Protein misfolding cyclic amplification reactions seeded with tissue homogenate from vCJD and control patients*

Tissue	Clinical vCJD patients, Met_129_/Met_129_					Preclinical vCJD patient, Met_129_/Val_129_		Non–vCJD controls, Met_129_/Met_129_	
	vCJD-1	vCJD-2	vCJD-3	vCJD-4		vCJD-A		NC-1	NC-2
Frontal cortex	10^−8^	10^−8^	10^−8^	10^−8^		–		–	–
Pituitary gland	NA	NA	NA	NA		10^−2^		–	–
Trigeminal ganglia	NA	NA	NA	NA		–		–	–
Dorsal root ganglia	NA	NA	NA	NA		–		–	–
Cervical lymph node	10^−5^	10^−4^	10^−4^	10^−3^		10^−4^		NA	NA
Tonsil	10^−3^	10^−4^	10^−6^	10^−3^		10^−3^		NA	–
Appendix	10^−4^	10^−4^	10^−4^	10^−3^		10^−2^		–	–
Distal Ileum	10^−3^	10^−5^	10^−5^	10^−2^		10^−3^		–	–
Spleen	10^−4^	10^−4^	10^−5^	10^−4^		10^−3^		–	–
Thymus	NA	10^−3^	10^−2^	10^−2^		10^−2^		NA	NA
Lung	10^−2^	10^−2^	–	–		10^−3^		–	–
Heart	10^−2^	10^−2^	–	–		–		–	–
Liver	10^−4^	10^−2^	10^−2^	10^−4^		10^−2^		–	–
Kidney	10^−2^	10^−3^	–	10^−3^		–		–	–
Salivary gland	10^−4^	10^−3^	10^−2^	10^−3^		10^−2^		–	–
Pancreas	10^−2^	–	10^−2^	10^−4^		–		–	–
Thyroid	10^−2^	–	10^−2^	10^−2^		–		–	–
Adrenal gland	10^−3^	10^−3^	10^−3^	10^−4^		–		–	–
Bone marrow	10^−4^	10^−5^	10^−3^	10^−4^		–		–	–
Skeletal muscle	10^−4^	10^−2^	–	NA		–		–	–
Testis	–	NA	–	10^−3^		NA		NA	NA
Ovary	NA	10^−4^	NA	NA		NA		NA	NA

*PMCA reactions were seeded with 10-fold serial dilutions of 10% tissues homogenates (10^−2^–10^−9^) that had been collected postmortem from 4 symptomatic vCJD patients (vCJD-1–vCJD-4) or an asymptomatic vCJD-infected person (vCJD-A). At least 4 replicates of each sample dilution were tested in 2 independent PMCA experiments. Prions from patients vCJD-1–vCJD-4 were homozygous for methionine at codon 129 of the *PRNP* gene. Prion from patient vCJD-A was heterozygous (methionine/valine) at codon 129 of the *PRNP* gene. PMCA substrate was prepared by using brains from transgenic mice overexpressing the ARQ variant of sheep prion protein. Reactions seeded with tissues from 2 non–vCJD-infected control patients (NC-1 and NC-2) were included as negative controls. PMCAs were subjected to 4 rounds of amplification, each composed of 96 cycles (sonication for 10 s and incubation for 14.5 min at 39.5°C) in a Qsonica700 Sonicator (Qsonica LLC, Newtown, CT, USA). PMCA reactions were analyzed by Western blotting for proteinase K–resistant PrP by using Sha31 antibody epitope YEDRYYRE. Values are the highest dilution that resulted in a positive Western blot result in >50% of the tested replicates after 4 PMCA amplification rounds. NA, not applicable; PMCA, protein misfolding cyclic amplification; PrP, prion protein; ­vCJD, variant Creutzfeldt-Jakob disease; –, negative.

We found that none of the reactions seeded with tissue homogenates from non–CJD controls were positive for PrP^res^ (Table 4). In contrast, PMCA reactions seeded with tissues from the 4 symptomatic vCJD patients were positive for PrP^res^ (Table 4; Figure 2). As expected, among tested tissues, brain homogenates (temporal cortex) showed the highest seeding activity (highest PrP^res^-positive dilution 10^−8^). All lymphoid organs tested also showed seeding activity, but the highest PMCA-positive dilution varied according to the organs tested from 10^−2^ (thymus) to 10^−6^ (distal ileum and tonsil). Moreover, for a given lymphoid organ, <10^2^-fold differences was observed in seeding activity, depending on the patient and sample tested. These data indicate that for symptomatic vCJD patients, lymphoid organs contain 10^2^–10^6^-fold less prion seeding activity than the same amount of brain tissue (Table 4).

**Figure 3:**
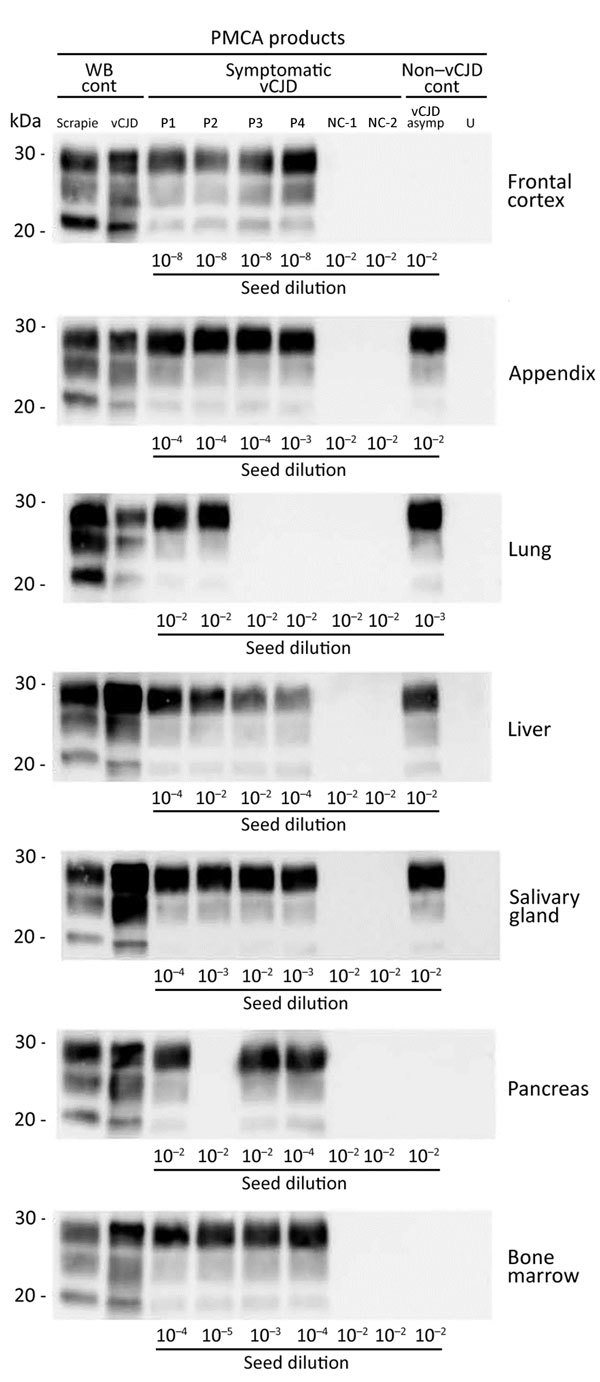
Western blots of proteinase K–resistant prions (PrP^res^) in PMCA reactions seeded with peripheral tissues. PMCA reactions were seeded with a 10-fold dilution series (10^−2^–10^−9^) of variant Creutzfeldt-Jakob disease (vCJD) tissue homogenates that had been collected postmortem from vCJD patients during the clinical stage (symptomatic vCJD patient 1–vCJD patient 4 [P1–P4]) or at an asymptomatic or preclinical stage of the disease (vCJD asymp) (Table 2). Reactions seeded with tissues from 2 non-vCJD patients (Table 2) were used as controls, and an unseeded (lane U) reaction was included as a specificity control. Reactions were then subjected to 4 amplification rounds, each composed of 96 cycles (sonication for 10 s and incubation for 14.5 min at 39.5°C) in a Qsonica700 Sonicator (Qsonica LLC, Newtown, CT, USA). PMCA reactions were analyzed by using Western blotting for abnormal PrP^res^ (Sha31 antibody epitope YEDRYYRE). A sheep scrapie sample and a vCJD reference isolate were used as controls. For the 7 tissues tested, the dilution of tissue homogenates used to seed the PMCA reactions is indicated below the immunoblots. Cont, control; WB, Western blot.

Salivary gland, adrenal gland, liver, and bone marrow from the 4 symptomatic vCJD patients showed positive reactions by PMCA (Figures 2, 3). Using the highest dilution to show a positive reaction as a measure of seeding activity, we found that the vCJD agent in these tissues was 10^3^–10^6^-fold lower than that for the brain. PrP^res^ was also detected by PMCA reactions seeded with heart, liver, kidney, skeletal muscle, several endocrine/exocrine glands (pancreas, thyroid), and gonads, from some, but not all, of the 4 clinical vCJD patients. Positive tissues contained a level of vCJD seeding activity that was equivalent to those observed in distal ileum (i.e., 10^3^–10^6^-fold lower than for the brain). Irrespective of the tissue used to seed the PMCA reactions, the PrP^res^ Western blot profile for positive reactions was indistinguishable from that observed in reactions seeded with the vCJD brain control (Figure 3).

### Analysis of Tissues from an Asymptomatic vCJD-Infected Person

Prion seeding activity was not detected in the brain (temporal cortex) of the asymptomatic vCJD–affected patient, who was infected with a *PRNP* gene codon 129 heterozygote (Met/Val_129_) prion (*12*) (Table 4; Figure 2). PMCA reactions seeded with dorsal root ganglia or trigeminal ganglia homogenates from this patient showed negative results. However, seeding activity was detected in the pituitary gland (highest PrP^res^-positive dilution 10^−2^). In addition, as for the symptomatic vCJD patient, PMCA amplification readily detected vCJD prions in all lymphoid organs tested from this asymptomatic person. On the basis of PMCA results, the vCJD agent load in lymphoid organs in this asymptomatic patient infected with the *PRNP* gene codon 129 Met/Val_129_ prion was similar to those for patients infected with Met/Met_129_ prions during the clinical stage of disease.

In addition to lymphoid organs, prion seeding activity was detectable in certain peripheral tissues (salivary gland, lung, and liver) from this patient (Tables 4; Figures 2, 3). Certain tissues, such as bone marrow or adrenal gland, that contained a substantial prion seeding activity in the clinically affected patients showed negative results. Again, the PrP^res^ Western blot profile for positive reactions was indistinguishable from that observed for reactions seeded with the vCJD brain control.

### vCJD Infectivity in Bone Marrow

**Figure 4:**
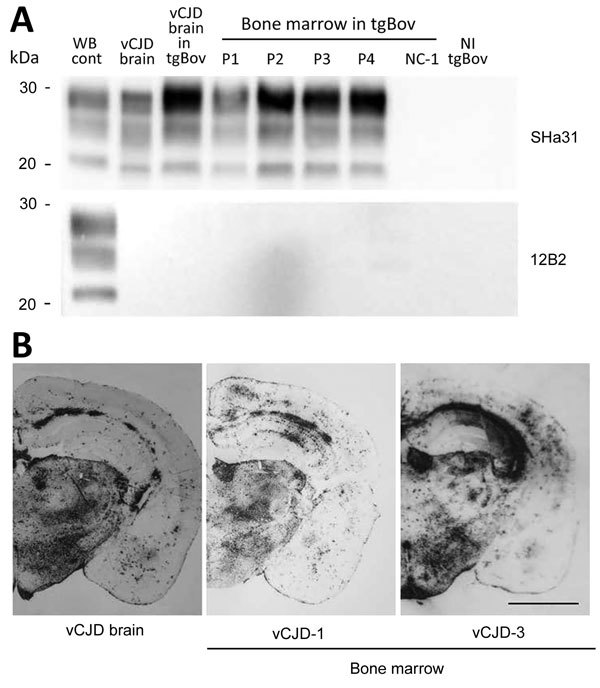
Detection of proteinase K–resistant prions (PrP^res^) by using Western blotting and paraffin-embedded tissue (PET) blotting of brains of transgenic mice expressing bovine PrP (tgBov). A) PrP^res^ WB of a vCJD sample (frontal cortex), tgBov mice (brain) inoculated with the same vCJD reference isolate, bone marrow samples from vCJD-affected patients (vCJD 1–vCJD-4 [P1–P4]; Table 2), and a non–vCJD control (NC-1; Table 2). A scrapie isolate (WB cont) and a noninoculated tgBov brain (vCJD brain) homogenate were included as controls. PrP^res^ immunodetection was performed by using Sha31 monoclonal antibody (epitope _145_YEDRYYRE_152_) and 12B2 epitope (epitope _89_WGQGG_93_). B) PET blotting of PrP^res^ distribution in coronal section (thalamus level) of tgBov mice inoculated with a reference vCJD isolate (10% brain homogenate) or bone marrow (10% tissue homogenate) from 2 vCJD patients (vCJD-1 and vCJD-3; Table 2) at the clinical stage of disease. Immunodetection of PrP^res^ was performed by using Sha31 monoclonal antibody (epitope _145_YEDRYYRE_152_). Cont, control; NI, not inoculated; WB, Western blot. Scale bar indicates 120 μm.

To test whether PMCA seeding activity in peripheral tissues from vCJD patients correlated with infectivity, we inoculated bone marrow samples from the symptomatic patient into tgBov mice. Clinical TSE was observed in mice that were inoculated with each of the 4 bone marrow samples. The PrP^res^ Western blot profile and the PrP^res^ distribution pattern, as assessed by paraffin-embedded tissue blotting for brain of the bone marrow–inoculated mice, were identical to those observed in tgBov mice inoculated with the vCJD brain control sample (Figure 4).

**Figure 5:**
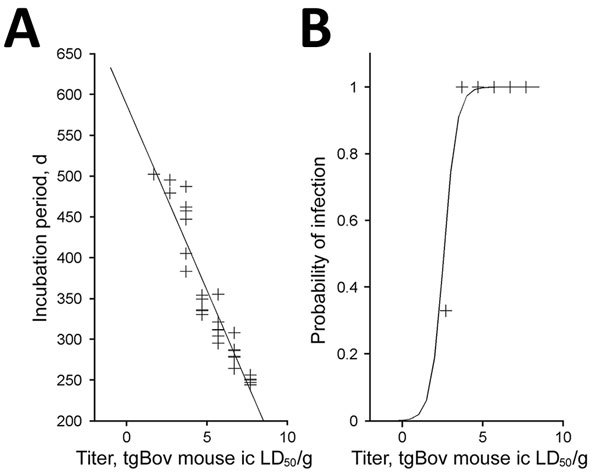
Dose–response relationship for A) incubation period and B) probability of infection of bovine PrP–expressing mice. Data were derived from an endpoint titration of 10% (wt/vol) frontal cortex homogenate from a patient with variant Creutzfeldt-Jakob disease. This homogenate was inoculated into tgBov mice (20 μL by intracerebral [ic] route; Table 1). This procedure was used to establish a model that estimates infectious titer in a homogenate on the basis of incubation period and the probability of infection in inoculated mice. Model plots are indicated by solid lines. +, observed value. LD_50_, 50% lethal dose.

Data obtained for mice inoculated with bone marrow samples were also used to estimate prion infectivity levels in these samples. For this purpose, we applied the method of Arnold et al. (*17*). This method combines the probability of survival (attack rate) and the individual mouse incubation period to provide an estimation of infectious titers. We used data corresponding to endpoint titration in tgBov mice for reference vCJD sample (frontal cortex from a clinical vCJD patient) (Table 1) to derive the relationship between prion titer of inoculum and the probability of infection and length of the incubation period (Figure 5). We found that bone marrow samples had an infectious titer that ranged from 10^2.3^ LD_50_/g through 10^4.7^ LD_50_/g in tgBov mice (Table 5).

**Table 5:** Bone marrow sample bioassay in bovine PrP–expressing mice (tgBov) for 4 patients with vCJD*

Sample	Transmission in tgBov mice		Mean infectious titer, LD_50_/g (95% CI)
	No. positive/no. inoculated mice	Mean ± SD incubation, d	
vCJD-1	5/5	458 ± 37	10^3.1^ (10^2.6^–10^3.5^)
vCJD-2	6/6	373 ± 35	10^4.7^ (10^4.3^–0^5.2^)
vCJD-3	4/6	504 ± 10	10^2.3^ (10^1.8^–10^2.7^)
vCJD-4	6/6	447 ± 91	10^4.0^ (10^3.4^–0^4.5^)
PBS control	0/6	>600	NA

*A 10% wt/vol bone marrow homogenate prepared from 4 symptomatic vCJD patients (Table 3) was inoculated intracerebrally into 6 tgBov mice (20 µL/mouse). One mouse (inoculated with homogenate from patient vCJD-1) died within the first few days after intercerebral inoculation. Mice were euthanized when they showed clinical signs of prion infection or after 600-d postinoculation. Mice were considered prion infected when abnormal PrP deposition was detected in brain. Infectious prion titers were estimated by using the method of Arnold et al. (*17*). The method uses the probability of survival (attack rate at each dilution) and the individual mouse incubation periods at each dilution to estimate infectious load. Infectious titers are given as estimated values. LD_50_, 50% lethal dose; NA, not applicable; PBS, phosphate-buffered saline; PrP; prion protein; vCJD, variant Creutzfeldt-Jakob disease.

These values are consistent with a 10^3^–10^5^ lower infectivity load in bone marrow samples than in the reference vCJD brain sample. Consistent with the PMCA results (Table 4), we found that prion load in bone marrow samples (highest PrP^res^–positive dilution [10^−3^–10^−5^]) was also 10^3^–10^5^-fold lower than for the reference vCJD isolate (highest PrP^res^–positive dilution [10^−8^]). These results strongly support the idea that PMCA seeding activity provides a reliable estimate of the prion load in tissues from vCJD-infected patients.

## Discussion

Most previous studies with tissue from vCJD patients have failed to identify consistent accumulation of the vCJD agent outside the nervous and lymphoreticular systems. However, data obtained in this study clearly demonstrate the presence of vCJD prions in a wide and unexpected variety of peripheral tissues.

Natural scrapie and experimental BSE in sheep are 2 models of orally transmitted prion diseases (*24*,*25*). In both diseases, the agent accumulates in the lymphoreticular system and the enteric nervous system during the early preclinical phase of the incubation period. Moreover, an early and persistent prionemia is observed in asymptomatic infected animals (*26*,*27*). These features were also observed in vCJD in humans and in view of the likely origin of vCJD (oral exposure to BSE agent), these similarities have led to a consensus that BSE and scrapie in sheep and vCJD in human have a common pathogenesis (*28*).

Although vCJD prions in a variety tissues, such as bone marrow, kidney, salivary gland, skeletal muscle, pancreas, liver, or heart, might be surprising, each of these tissue has already been demonstrated to accumulate prion infectivity or abnormal prion protein in TSE-infected sheep (*29*–*33*). Because low levels of infectivity have been reported in blood fractions from a vCJD-affected patient, such widespread tissue positivity might be derived from residual blood, rather than from the solid tissue in these samples (*16*). However, this proposal seems unlikely because in whole blood PMCA amplification inhibitors preclude detection of endogenous vCJD agent by this method (*11*,*34*–*36*).

The patient in our study who was infected with a prion containing *PRNP* gene codon 129 Met/Val is 1 of only 2 identified vCJD agent–infected persons known to have died of other causes before onset clinical symptoms of vCJD, and the only person who provided consent to sample autopsy tissues for research. For this patient, all previous investigations did not detect abnormal prion protein or infectivity in the brain (*12*,*37*). The negative PMCA results we obtained for cerebral cortex, dorsal root ganglia, and trigeminal ganglia tissue from this patient are consistent with a lack of central nervous system involvement at the time of death. However, PMCA seeding activity in the pituitary gland was surprising in this instance.

The presence of abnormal prion protein accumulation in the pituitary gland and other circumventricular organs before deposition of PrP^res^ in surrounding brain has been reported in TSE-infected sheep (*38*). However, this phenomenon in animals does not represent the main route for neuroinvasion and is a probable consequence of hematogenous dissemination of the TSE agent through the fenestrated capillary system of the circumventricular organs, which is substantially more permeable than the other capillaries in the brain (blood–brain barrier). Therefore, this finding might be a consequence of the hematogenous route of secondary vCJD in this person (by transfusion of packed erythrocytes from a vCJD-infected donor), in contrast to the oral route of infection in primary clinical vCJD cases (*12*).

vCJD prions were detected in certain peripheral tissues from the patients infected with a prion containing the *PRNP* gene codon 129 Met/Val. Although distribution of vCJD seeding activity in lymphoreticular tissues was similar to that observed for symptomatic vCJD patients, several tissues that were positive in clinically affected patients were negative in this heterozygous asymptomatic person. These findings suggest that involvement of some peripheral tissues might occur at a later stage in the incubation period than others, or that they could involve recirculation of the agent from the central nervous system (i.e., centrifugal spread in a late state). However, we cannot discount the possibility that that these differences in tissue distribution are caused by the hematogenous route of infection in this person (as opposed to the probable oral route in patients with clinical vCJD) or the difference between the *PRNP* gene codon 129 genotype of the asymptomatic vCJD–affected person (*PRNP* gene codon 129 Met/Val) and persons with clinical vCJD (*PRNP* gene codon 129 Met/Met).

Irrespective of the actual explanation for these differences, the presence of vCJD agent in peripheral tissues of patients during preclinical and clinical stage of the disease indicates the potential for iatrogenic transmission of this fatal neurologic condition by surgical procedures. Furthermore, this finding shows that, for certain peripheral tissues, a level of infectivity equivalent to an end stage titer (and attendant risk) is reached at a preclinical stage.

Several hundred cases of iatrogenic CJD have been reported worldwide. These cases appear to result from transmission of sporadic CJD, and most cases have occurred in recipients of human dura mater grafts or after administration of human growth hormone extracted from cadaveric pituitaries (*39*). Although in sporadic CJD the distribution of the agent is largely restricted to the nervous system (central and peripheral), the wide distribution of the vCJD agent in the asymptomatic infected patient we report might serve to increase the range of medical procedures, including dentistry, organ transplant, and surgery involving nondisposable equipment, that might result in iatrogenic transmission of vCJD (*40*–*43*).

Nevertheless, >20 years after identification of the first vCJD patients, only 5 cases that are a probable consequence of iatrogenic vCJD transmission are known, all in the United Kingdom and associated with blood and blood products. These cases were caused by transfusion of non–leukocyte-depleted erythrocyte concentrates or by treatment involving large amounts of pooled plasma from the United Kingdom that were known to include donations from persons who later showed development of vCJD (*12*,*44*–*46*).

None of the 220 other vCJD cases identified worldwide have been linked to any other medical or dental procedure. Whereas this fact is reassuring, it would be unwise to disregard the threat that vCJD still poses for public health. Despite the relatively low number (n = 178) of vCJD clinical cases observed in the United Kingdom, the most recent epidemiologic studies indicate that ≈1 of 2,000 persons in the United Kingdom could be infected with the vCJD agent (as indicated by the presence of abnormal prion protein detected by immunohistochemical analysis of lymphoid follicles in the appendix). Each asymptomatic vCJD-infected person represents a potential source of secondary infection. The data in our report offer an opportunity for refining measures that were implemented in many countries to limit the risk for vCJD iatrogenic transmission. The apparent concordance between PMCA biochemical and infectivity bioassay data, and the higher analytical sensitivity of PMCA, suggest that future research need not rely exclusively on time-consuming and costly animal bioassay.

Our results indicate the need for vCJD screening assays. After more than a decade of effort, several vCJD blood detection tests have reached a stage in their development that could enable their evaluation as screening or confirmatory assays (*11*,*47*,*48*). In particular, there is now a strong case for use of PMCA in a highly sensitive and specific blood test for vCJD, as indicated by our previous studies (*11*,*16*) and studies by Bougard et al. (*35*) and Concha-Marambio et al. (*36*). The relationship shown here between PrP^res^ amplification by PMCA and detection of infectivity by bioassay indicates that PMCA seeding activity is a good surrogate marker of infectivity and could provide a sound basis for a vCJD blood test for use with blood or tissue donors.
